# Change in hearing loss and its association with general health in a community-based longitudinal study of middle-aged individuals: The Busselton healthy ageing study

**DOI:** 10.1371/journal.pone.0360651

**Published:** 2026-10-15

**Authors:** Adriana L. Smit, Robert H. Eikelboom, Yinan Mao, Michael Hunter, Marcus D. Atlas, De Wet Swanepoel, Inge Stegeman

**Affiliations:** 1 Department of Otorhinolaryngology, Head & Neck Surgery, University Medical Center Utrecht, Utrecht, Netherlands; 2 UMC Utrecht Brain Center, University Medical Center Utrecht, Utrecht, Netherlands; 3 Centre for Ear Sciences, The University of Western Australia, Nedlands, Australia; 4 Department of Speech Language Pathology and Audiology, University of Pretoria, Pretoria, South Africa; 5 Ear Science Institute Australia, Subiaco, Australia; 6 School of Population and Global Health, University of Western Australia, Perth, Australia; 7 Busselton Health Study Centre, Busselton Population Medical Research Institute, Busselton, Australia; 8 Department of Otolaryngology-Head and Neck Surgery, University of Colorado School of Medicine, Aurora, Colorado, United States of America; LSU Health Shreveport, UNITED STATES OF AMERICA

## Abstract

**Introduction:**

With the ageing of the global population, disabling hearing loss is a major health concern. While age and sex are established determinants, the relation between general and environmental health factors and the progression of hearing loss remains incompletely understood, particularly in middle-aged adults.

**Methods:**

We used data from the *Busselton Healthy Ageing Study (BHAS)*, a population-based cohort in Western Australia. Adults aged 45–70 years at baseline (2010–2015) underwent audiometric testing and health assessments with a six-year follow-up. The better-ear four-frequency Pure Tone Averages for 0.5-4kHZ (4FPTA) and for high frequencies (4 & 8 kHz) (HFPTA) were calculated. Hearing loss was classified according to WHO criteria. Demographic, ear-related, general health, and environmental factors were analysed and their association with change in hearing thresholds using linear mixed models.

**Results:**

3596 participants with reliable audiometry at baseline and follow-up were included (mean baseline age 57.9 years, 55% female). Over 6 years follow-up, the prevalence of mild-or-greater hearing loss increased from 14% to 26% (4FPTA) and from 53% to 71% (HFPTA). Thresholds worsened across all age categories, with greater deterioration in men and at higher frequencies. In univariate models, older age, male sex, higher BMI, smoking, tinnitus, chronic ear infection, noisy workplace exposure (particularly with inconsistent or absent hearing protection), hypertension and pre-diabetes were significantly associated with faster hearing decline after adjustments for age and sex.

**Conclusions:**

In this longitudinal population-based study of middle-aged Western Australians we demonstrated that hearing thresholds significantly declined over a six-year period with greater deterioration in men and at higher frequencies. Findings suggest that hearing loss progression in midlife is shaped not only by age and sex, but also by broader metabolic and environmental health profiles. These outcomes highlight the need for future research to further clarify the complex interplay between metabolic health, environmental exposures, and hearing outcomes.

## Introduction

With the rise and ageing of the global population, the increasing numbers of individuals living with disabling hearing loss is considered one of the most important global health problems according to the WHO [1]. Hearing loss can have a major impact on daily life by disturbing communicative abilities but is also implicated in social isolation, depression, cognitive decline and dementia [2]. Identifying and understanding factors that contribute to the onset and progression of hearing loss in middle-aged adults could influence patient counseling, rehabilitation and possible treatment of the underlying condition

The prevalence of hearing loss increases significantly with age and even accelerates with age [3–5]. Furthermore, different patterns of hearing deterioration can be seen between males and females [6]. Currently, underlying mechanisms of hearing loss across age are only partly understood. Besides physiological degeneration of the inner ear structures, health-related factors as well as environmental and genetical factors have their effect on hearing deterioration over time [7,8].

To date, most studies focusing on the identification and quantification of factors contributing to age-related hearing loss have been based on cross-sectional studies, and findings have been inconsistent. This can be explained by the differences in measures of risk factors (e.g., based on self-reported medical history of hypertension in contrast to objective blood pressures measures of hypertension), hearing outcomes (e.g., subjective assessment of hearing handicap versus objective assessment of hearing levels by pure tone audiometry), study setting (e.g., visitors to ear clinics versus population studies) and/or characteristics of the participants (e.g., age range). Instead, longitudinal studies allow assessment of temporal relationships and are therefore better suited to determine whether potential risk factors precede changes in hearing. Additionally, population studies can contribute to determining which factors are involved in the etiology of hearing loss. This knowledge could contribute to identifying potentially modifiable factors, which in turn may help to reduce or delay age-related hearing loss and its broader impact on society.

Only a few longitudinal studies have investigated the association between demographics, health-related, and environmental risk factors and objectively measured changes in hearing levels over time in population-based settings. *Demographic factors* were studied in the EHLS (the Epidemiology of Hearing Loss Study, USA as a follow-up study on the Beaver Dam Eye study) with a follow-up up to 15 years. In addition to older age being a risk factor for both the incidence and progression of hearing loss, male sex and indicators of lower socioeconomic status were also found to be associated with a higher incidence of hearing loss, even after adjusting for age [9–11]. A history of noisy occupation was not found to be associated [10]. In a longitudinal analysis of 1436 adults aged 18–89 + years from a US community-based cohort, average hearing thresholds declined by 0.83 dB per year (95% CI 0.79–0.86), with females (versus males) showed higher rates of threshold change in higher frequencies but less decline per year in lower frequencies [12]. A Dutch population-based cohort, with a follow-up of 12 years, also found that higher age and male gender were significantly associated with poorer baseline hearing thresholds and faster deterioration of hearing loss [13]. An Australian population study, with a follow-up period of 11 years, also found that men had higher baseline hearing thresholds, but women experienced faster rates of decline in hearing for mid- to high-range frequencies [6]. These findings were not confirmed in a Dutch population study with a shorter follow up period (4 years), in which higher age was associated with worse hearing thresholds at baseline, but there was no statistical evidence that the tested determinants such as sex, age and education level were associated with progression of hearing loss (Rigters et al. 2018).

Several other population-based studies have examined the association between *cardiovascular risk factors* and the progression of hearing loss over time using combinations of medical history taking and objective measures to assess the cardiovascular determinants. In a recent study, as part of the Canadian Longitudinal Study of Ageing, hypertension and the general cardiovascular risk profile score were associated with greater loss of hearing over the 3-year follow-up period for both sexes [14]. In addition, smoking in males and obesity in females were associated with faster rates of hearing decline over 3-year follow-up [14]. In a Dutch population-based cohort study with a follow-up of 12 years it was found that a large waist circumference was also associated with hearing deterioration over time in participants aged 24–42 years, but not in older age groups [13]. In the same study cardiovascular disease (CVD) was associated with faster hearing deterioration, but the effect was small and was found only in middle-aged (43–62 years) adults whereas obesity was associated with faster hearing loss in older adults only (age 63–81 years). These findings were not disaggregated by sex. Fifteen-year follow-up data from the EHLS concluded that current smoking, waist circumference and poorly controlled diabetes mellitus were associated with greater risk of incident hearing loss. Former smokers and people with better-controlled diabetes mellitus were not at greater risk [9]. In other population based longitudinal studies, smoking and diabetes [15], a history of stroke or diabetes [6], CVD [16] and persistent hypertension compared to normotension [17] were not confirmed to be associated with a change in hearing over time. Besides tobacco smoking, other *lifestyle factor*s such as alcohol consumption have scarcely been studied; Rigters et al found no association between alcohol consumption and the 4-year progression of hearing loss [15].

To clarify which factors contribute to hearing deterioration in midlife, using objective measures of hearing and health-related factors, this study investigates the trajectory of hearing thresholds over time, and secondly assesses associations between demographic, health-related and environmental characteristics and the progression of hearing loss in a population-based sample of middle-aged men and women.

## Materials and methods

This study is reported according to the STROBE statement (see S1 Checklist) [18].

### Study sample and recruitment process

This study used data from the Busselton Healthy Ageing Study (BHAS), which commenced in 2010 with the aim to identify the cumulative effects of multimorbidity that impacts on healthy aging. The BHAS includes detailed assessments of physical function, cognitive performance, and quality of life in baby boomers. Adults born between 1946 and 1964, residing in the City of Busselton, Western Australia, and listed on the electoral roll in the region, were invited to participate in this population-based study (6,690 individuals). Invitations to participate were issued in a randomized order, with recruitment efforts focused on sequential 10% sample draws. Of the 6690 adults invited, 5107 agreed to participate (response rate 76.3%). Recruitment for the cohort began in 2010. The baseline or first wave of data (“T0”) was collected between May 2010 and December 2015, resulting in a cohort aged 45–70 years. The first wave of follow-up (“T1”) data of these participants was collected between 2016 and 2021. For the current study only participants who completed audiometric tests at T0 and T1 were included.

This article focuses on the methods for this study. The full protocol of the study, including all measurements, is described elsewhere [19]. The study was conducted according to the Declaration of Helsinki and was approved by The University of Western Australia Human Research Ethics Committee (Number RA/4/1/2203). All participants gave their informed written consent before inclusion in the study.

### Outcome assessment

Data on demographics, general health and environmental factors were collected using a self-administered clinical history questionnaire that was completed by all participants who attended the testing centre. Audiometric hearing thresholds, objective measures of general health, including anthropometry and blood pressure, were gathered from physical assessments at the study centre. During this visit, a fasting venous blood sample was collected for biochemistry testing and analysed for biochemical measures [20]. For the current study, data on demographics, general health and environmental factors were based on the data from the baseline (T0) visit. The only data used from the follow-up visit (T1) were those related to hearing thresholds.

### Demographics

Demographic data included sex and age at the time of first assessment. Based on this variable age groups were created based on 5-year intervals (45–50, > 50–55, > 55–60, > 60–65, > 65–70 years of age). Highest education level obtained was scored (no school, primary, secondary school, other (e.g., technical school, college) or university) as well as the average annual total household income in Australian dollars (AUD).

### Ear-related health variables

#### Hearing outcome assessment.

Hearing levels were determined at T0 and T1 by pure tone air and bone conduction threshold measurements using automated audiometry with AMTAS software (AMTAS, Audiometry Incorporated, USA) controlling a Conera audiometer (GNOtometrics, Copenhagen, Denmark), with Sennheiser HDA 200 (Wennesbostel, Germany) headphones, on a NOAH platform (Hearing Instrument Manufacturers’ Software Association, Copenhagen, Denmark) and conducted in a sound-proof booth (Acoustic Designs, Australia). Quality control of the testing procedures was guaranteed by (i) training of the technical staff, and (ii) the use of Qualind quality assessment methods built into the automated audiometer software [21]. The testing protocol was in accordance with Hughson-Westlake methods (James et al). Audiometry was supervised by a trained nurse, who instructed the participant, fitted the headset, started the test, and ensured that the data were saved. She/he did not have any training in assessing the quality of the test. The automated audiometer system incorporates a system of grading the quality of the test: as “good”, “fair”, “poor” or “fail’. For the study, only participants with a complete hearing assessment at both T0 and T1 and with automated test-quality ratings of “good” or “fair” at both time points were included.Air conduction thresholds were recorded at 250, 500, 1,000, 2,000, 4,000, and 8,000 Hz. The four-frequency Pure Tone Averages (PTA) for both ears were calculated as the mean of the air conduction hearing levels for 500, 1,000, 2,000, and 4,000 Hz; the lowest average was considered as the better ear (4FPTA). Similarly, the best ear high frequency pure tone average (HFPTA) was calculated by taking the mean of the air conduction hearing levels for 4,000 and 8,000 Hz. These 4FPTA and HFPTA of the best ear were used to categorize hearing loss in scales of impairment based on the WHO classification (no impairment < 20 dB; mild 20–34 dB; moderate 35–49 dB; moderately severe 50–64 dB; severe 65-79dB; profound impairment 80–94 dB) [22].

#### Tinnitus.

Participants reported the presence of tinnitus by the question “Do you experience tinnitus (sound in your ears and head) for longer than 5 minutes which does not have an obvious cause (no, yes)?”.

#### Chronic ear infection.

Participants were asked if they could indicate whether they were ever told by their doctor that they had chronic ear infection (yes, no).

#### Noise exposure.

Noise exposure was determined by: Q1 “*Have you worked in a place where it was so noisy that you had to raise your voice to be heard by others (no, yes)*?”; if answered ‘yes’, then the frequency was asked (Q2) (never, occasionally, frequently, always). Workplace noise level assessment was classified as: ‘Quite workplace’ (Q1 no), ‘Noisy workplace - Always protected’ (Q1 yes, Q2 always), ‘Noisy workplace - Frequently protected’ (Q1 yes, Q2 frequently), ‘Noisy workplace - Occasionally protected’ (Q1 yes, Q2 occasionally), ‘Noisy workplace - Never protected’ (Q1 yes, Q2 never).

### General health variables

#### Hypertension.

Blood pressure was measured with the participant supine, after five minutes of rest. Systolic and diastolic blood pressures (MAP) were measured using a vascular profiler (Omron VP1000). Blood pressure was scored in stages according to the classification of the American Heart Association (AHA). These stages are based on systolic (SBP) and diastolic blood pressure (DBP) levels into normal (SBP < 120 mm Hg & DBP < 80 mm Hg), elevated (SBP ≥ 120 to <130 mm Hg & DBP < 80 mm Hg), hypertension stage 1 (SBP ≥ 130 to <140 mm Hg or DBP ≥ 80- < 90 mm Hg) or hypertension stage 2 (SBP ≥ 139 mm Hg or DBP > 89 mm Hg) [23].

#### Cardiovascular disease.

Participants were categorized as having a heart disease if they reported any of the following conditions/situations: angina, having a cardiac pacemaker/implant, myocardial infarction, coronary bypass or stent. A stroke was scored if participants indicated that they were told by their doctor that the patient had a transient ischemic attack (TIA), stroke or underwent carotid surgery. Peripheral artery disease (PAD) was indicated by an ankle brachial index (ABI) at the left or right of <0.9, or if ABI was missing, by a positive history of having claudication. Subsequently, if participants were indicated as having a heart disease, or stroke or peripheral artery disease based on above mentioned criteria, they were categorized as having cardiovascular disease.

#### Body mass index.

Standing height and weight were measured with the participant lightly clothed and shoeless using standard anthropometric techniques. Body Mass Index (BMI) was calculated based on height and weight and reported as a continuous outcome. Subsequently, BMI was categorized into <18.5 kg/m2 underweight, 18.5–24.9 kg/m2 healthy weight, 25.0–29.9 kg/m2 overweight, > 30 kg/m2 obese [18].

#### Diabetes.

A fasting venous blood sample was collected for biochemistry testing. The outcomes included general chemistry of plasma (Fasting Plasma Glucose; FPG) and whole blood (glycated haemoglobin; HbA1c). The criteria of the American Diabetes Association (ADA) 2019 were used to classify participants as having diabetes or pre-diabetes based on FPG and HbA1c outcomes (diabetes; glycated haemoglobin > 6.5% or fasting plasma glucose > 7.0 mmol/L®; pre-diabetes; glycated haemoglobin 5.7–6.4% or fasting plasma glucose 5.6–6.9 mmol/L) [24].

#### Smoking.

Pack years of smoking was calculated based on the outcomes of two questions:” Have *you ever smoked cigarettes?”* (No, Yes). In case of ‘Yes’, participants were asked how many cigarettes per day they smoked and the number of years that they reported smoking. The duration of smoking in years was calculated based on the age reported by participants that they started and stopped smoking. Based on these, numbers of pack years of smoking per participant were calculated in which one pack years was defined as 20 cigarettes per day for a year. Pack years were described as a continuous variable as well as in categories of smoking pack years; 0 pack years = never smokers / 0.01–20 pack years = light smokers/ 20.01–40 pack years = moderate smokers / more than 40 pack years = heavy smokers.

#### General health score.

Self-reported general health was scored by the outcomes of a single question;” *In general, would you say your health is:”* answered as ‘excellent’, ‘very good’, ‘good’, ‘fair’ or ‘poor’.

### Statistical analyses

For the hearing measurements, records with unreliable audiograms of participants, i.e., those with “Poor” or “Failed” reliability as judged by the automated audiometer software, were excluded from the analysis. Median hearing levels were reported descriptively per audiometric test frequency and age categories for T0 and T1 for the whole group and sexes separately. Demographic variables and measures of general and environmental factors of the studied population were reported. To describe variables, mean and standard deviation (SD) were used for continuous variables, count number and percent were used for categorical variables.

Missing data were anticipated in covariates included in subsequent analyses. Missing data were handled using multivariate imputation by chained equations. The mice package in R was used to generate five imputed datasets, using predictive mean matching as the imputation method, a maximum of 10 iterations, and a random seed of 12345. Analyses were performed separately within each imputed dataset, and parameter estimates and standard errors were subsequently pooled across the five imputed datasets using Rubin’s rules.

Linear mixed-effects models with repeated measures were used to assess associations between demographic, health-related, and environmental factors and changes in hearing thresholds between baseline (T0) and follow-up (T1). Models were fitted separately for each explanatory variable. First, unadjusted univariate models were fitted, with each covariate entered individually as the fixed effect. Second, the univariate models were repeated with adjustment for age and sex, where applicable, because the age and sex distribution of the study sample differed from that of the general Busselton population, based on Census data from the Australian Bureau of Statistics [25]

Demographic variables included age or age group and sex. General health measures included history of cardiovascular disease and chronic ear infection as dichotomous variables; diabetes status, blood pressure category, and general health score as categorical variables; and BMI and smoking pack-years as continuous variables. Occupational noise exposure in combination with hearing protection use was included as a categorical environmental variable.

All models were fitted using the lmer() function from the lme4 package in R with default estimation settings. A participant-level random intercept was included to account for repeated observations within individuals. No random slopes or additional residual covariance structures were specified; therefore, within-participant correlation was accounted for through the random intercept. Conditional on the fixed and random effects, residuals were assumed to be independent and to have constant variance. Model assumptions included normally distributed residuals and random effects, linear associations for continuous covariates, independence between participants, and homoscedasticity. These assumptions were evaluated using standard diagnostic plots.

Outcomes are based on univariate models and provided in β and 95% confidence intervals. R package *emmeans* was used to extract the effects. A value of p < 0.05 was used to define a statistically significant outcome.

## Results

### Characteristics of studied sample

3679 participants completed both T0 and T1 measurements. Of those, 22 participants (0.6%) could not be matched across the two phases. Of the 3657 participants remaining, 18 participants at T0 and 43 at T1 were excluded based on insufficient hearing test quality (T0: 16 audiograms rated as “Poor” and 2 as “Fail”/ T1: 24 rated as “Poor” and 19 as “Fail”). Therefore, 3596 (97.7%) participants remained for analysis.

A total of 26 (0.72%) records contained missing values among the variables of interest and were imputed using multivariate imputation by chained equations.

All relevant characteristics of the study population at T0 are displayed in Table 1.

**Table 1:** Characteristics of the studied population.

Variable	Category	N (%)		Variable	Category	N (%)
Demographics				General health		
Sex	Male	1609(45%)		Blood pressure	Normal	877(24%)
	Female	1987(55%)			Elevated	713(20%)
Age (years)	(45,50]	385(11%)			Hypertension I	1542(43%)
	(50,55]	870(24%)			Hypertension II	464(13%)
	(55,60]	932(26%)			NA	0(0%)
	(60,65]	981(27%)		Cardiovascular diseases	No	1945(54%)
	(65,70]	428(12%)			Yes	1651(46%)
Income (AUD)	Less than $20,000	187(5%)		Diabetes	No	1806(50%)
	$20,001 to $40,000	553(15%)			Pre-DM	1648(46%)
	$40,001 to $60,000	618(17%)			DM	142(4%)
	$60,001 to $80,000	479(13%)		General health score	Poor	23(1%)
	$80,001 to $100,000	458(13%)			Fair	248(7%)
	More than $100,000	857(24%)			Good	135(38%)
	NA	444(12%)			Very good	1470(41%)
Education	Primary school	34(1%)			Excellent	501(14%)
	Secondary school	1690(47%)			NA	1(0%)
	Other	1137(32%)		Smoking pack-year	Never smokers	1800(50%)
	University	730(20%)			Light smokers	1318(37%)
	NA	5(0%)			Moderate smokers	341(9%)
Ear-related health					Heavy smokers	137(4%)
Noisy exposure	Quiet workplace	2128(59%)		BMI	Normal	935(26%)
	Noisy-workplace-Always protected	151(4%)			Underweight	16(0%)
	Noisy-workplace- Frequently protected	322(9%)			Overweight	1556(43%)
	Noisy-workplace- Occasionally protected	552(15%)			Obese	1032(29%)
	Noisy-workplace- Never protected	439(12%)			NA	57(2%)
	Noisy workplace- NA	4(0%)				
Tinnitus	No	2781(77%)				
	Yes	815(23%)				
Chronic ear infection	No	3369(94%)				
	Yes	227(6%)				

Characteristics of the studied population of 3596 participants at T0. Demographic, ear-related and health-related outcomes are provided N (%).

At baseline, the mean age (SD; range) was 57.9 (5.7; 45.5–69.8) years (males 58.0 (5.77; 45.5–69.8), and 55% (1987 out of 3596) were females. The mean age (range) at follow-up was 64.0 (51.4–75.6) years, i.e., median of 5.9 (0.9) years (mean 6.2(0.9) years) between T0 and T1.

Regarding ear-related health, 815 participants (23%) were classified as having tinnitus, and 227 (6%) had been diagnosed with chronic ear infections. A total of 1,468 of 3,596 participants (41%) reported having worked in a noisy workplace. Among all participants, 552 (15%) reported occasionally wearing hearing protection in such environments, while 439 (12%) reported never using it.

With respect to general health, 1,790 participants (50%) were identified as having (pre)diabetes mellitus, and 1,986 (55%) had been diagnosed with some form of hypertension. Self-rated general health was reported as fair or poor by 271 participants (8%).

### Hearing outcomes based on hearing levels

The median 4FA PTA hearing level at baseline (T0) was 5 dB (10dB for males, and 5dB for females) and 10dB at follow-up (T1) (10dB for males, and 10dB for females). For the high frequences (HF PTA) this was 20 dB (25dB for males, and 15dB for females) and 30dB at follow-up (T1) (40dB for males, and 20 dB for females).

Pure-tone thresholds at baseline were worse for higher frequencies than for lower frequencies for men, for women and for both groups together. Thresholds from T0 to T1 worsened for each age category (See Table 2 and Figs 1–2).

**Table 2:** Change in hearing thresholds.

Sex	Age category (years)	N (%)	250 Hz (median, 95% CI)	500 Hz (median, 95% CI)	1000 Hz (median, 95% CI)	2000 Hz (median, 95% CI)	4000 Hz (median, 95% CI)	8000 Hz (median, 95% CI)	4FA (median, 95% CI)	HFA (median, 95% CI)
All	(45,50]	385 (11%)	0.0 (−10.5, 11.1)	0.0 (−11.0, 11.3)	0.0 (−8.3, 11.3)	5.0 (−10.6, 17.1)	5.0 (−11.6, 22.5)	5.0 (−15.3, 31.4)	2.5 (−5.9, 11.1)	5.0 (−10.0, 23.5)
	(50,55]	870 (24%)	0.0 (−19.2, 20.0)	0.0 (−14.1, 15.0)	0.0 (−10.3, 13.6)	5.0 (−9.0, 17.4)	5.0 (−10.6, 23.2)	7.5 (−15.0, 32.4)	2.5 (−6.4, 12.8)	7.5 (−9.3, 24.3)
	(55,60]	932 (26%)	0.0 (−22.0, 24.9)	0.0 (−17.4, 20.3)	0.0 (−11.2, 15.3)	5.0 (−13.5, 25.3)	5.0 (−12.9, 28.1)	10.0 (−16.5, 38.5)	3.8 (−8.7, 17.2)	7.5 (−10.7, 29.2)
	(60,65]	981 (27%)	0.0 (−20.2, 23.4)	0.0 (−13.0, 16.9)	0.0 (−9.7, 15.5)	5.0 (−8.5, 20.2)	5.0 (−10.3, 25.8)	10.0 (−13.3, 35.9)	3.8 (−5.1, 14.3)	7.5 (−7.6, 26.6)
	(65,70]	428 (12%)	0.0 (−29.0, 33.8)	0.0 (−22.5, 24.5)	0.0 (−13.6, 18.8)	5.0 (−10.8, 25.3)	5.0 (−16.1, 31.4)	10.0 (−17.1, 38.0)	3.8 (−9.4, 18.6)	7.5 (−12.6, 30.8)
	all	3596 (100%)	0.0 (−21.0, 23.5)	0.0 (−15.7, 18.0)	0.0 (−10.7, 15.1)	5.0 (−10.8, 21.5)	5.0 (−12.1, 26.3)	10.0 (−15.4, 35.7)	3.8 (−7.2, 15.1)	7.5 (−9.8, 27.1)
Female	(45,50]	211 (11%)	0.0 (−9.6, 10.8)	0.0 (−10.4, 11.5)	0.0 (−8.0, 11.6)	5.0 (−10.5, 16.5)	5.0 (−9.0, 18.6)	5.0 (−14.2, 27.8)	2.5 (−5.1, 10.2)	5.0 (−9.1, 20.7)
	(50,55]	499 (25%)	0.0 (−17.8, 16.6)	0.0 (−14.1, 15.1)	0.0 (−9.6, 13.5)	5.0 (−8.2, 15.9)	5.0 (−9.4, 19.8)	5.0 (−17.0, 31.6)	2.5 (−5.6, 11.3)	5.0 (−10.1, 22.6)
	(55,60]	521 (26%)	0.0 (−21.6, 25.5)	0.0 (−17.1, 21.3)	0.0 (−12.7, 16.6)	5.0 (−15.8, 27.6)	5.0 (−14.1, 28.9)	10.0 (−15.2, 37.8)	3.8 (−10.3, 18.9)	7.5 (−10.9, 29.6)
	(60,65]	535 (27%)	0.0 (−19.7, 22.3)	0.0 (−12.4, 16.3)	0.0 (−9.4, 14.5)	5.0 (−8.4, 19.2)	5.0 (−11.1, 26.4)	10.0 (−12.7, 36.2)	3.8 (−5.8, 14.5)	7.5 (−8.1, 27.5)
	(65,70]	221 (11%)	0.0 (−33.5, 38.8)	0.0 (−23.7, 26.4)	0.0 (−12.4, 16.9)	5.0 (−13.3, 26.8)	5.0 (−19.9, 36.9)	10.0 (−19.0, 39.2)	3.8 (−11.8, 21.2)	7.5 (−15.9, 34.5)
	all	1987 (100%)	0.0 (−21.3, 23.4)	0.0 (−15.6, 18.4)	0.0 (−10.6, 14.8)	5.0 (−11.7, 21.8)	5.0 (−12.9, 26.4)	10.0 (−15.7, 35.3)	3.8 (−8.0, 15.7)	7.5 (−10.8, 27.4)
Male	(45,50]	174 (11%)	0.0 (−11.5, 11.4)	0.0 (−11.6, 11.1)	0.0 (−8.7, 11.0)	5.0 (−10.7, 17.9)	5.0 (−14.0, 26.5)	10.0 (−16.1, 35.2)	2.5 (−6.8, 12.2)	7.5 (−10.6, 26.4)
	(50,55]	371 (23%)	0.0 (−20.3, 23.9)	0.0 (−14.2, 14.9)	0.0 (−11.1, 13.8)	5.0 (−9.8, 19.2)	5.0 (−11.5, 27.0)	10.0 (−11.8, 33.0)	3.8 (−7.4, 14.5)	7.5 (−7.7, 26.0)
	(55,60]	411 (26%)	0.0 (−22.5, 24.2)	0.0 (−17.6, 18.9)	0.0 (−9.0, 13.5)	5.0 (−10.1, 22.0)	5.0 (−11.3, 26.9)	10.0 (−18.2, 39.3)	3.8 (−6.4, 14.8)	7.5 (−10.4, 28.8)
	(60,65]	446 (28%)	0.0 (−20.7, 24.7)	0.0 (−13.6, 17.5)	5.0 (−10.0, 16.6)	5.0 (−8.6, 21.3)	5.0 (−9.4, 25.1)	10.0 (−14.1, 35.4)	5.0 (−4.3, 14.1)	7.5 (−7.0, 25.5)
	(65,70]	207 (13%)	0.0 (−23.3, 27.6)	0.0 (−21.1, 22.3)	0.0 (−14.8, 20.7)	5.0 (−7.9, 23.3)	5.0 (−10.5, 24.2)	10.0 (−15.0, 36.8)	3.8 (−6.3, 15.4)	7.5 (−8.4, 26.1)
	all	1609 (100%)	0.0 (−20.8, 23.7)	0.0 (−15.8, 17.5)	0.0 (−10.8, 15.3)	5.0 (−9.6, 21.1)	5.0 (−11.0, 26.1)	10.0 (−15.0, 36.1)	3.8 (−6.2, 14.4)	7.5 (−8.6, 26.7)

Change in median thresholds in dB between T0 and T1 (with 95%CI) for each test frequency per ear, for females, males and both, by age group.

**Fig 1:**
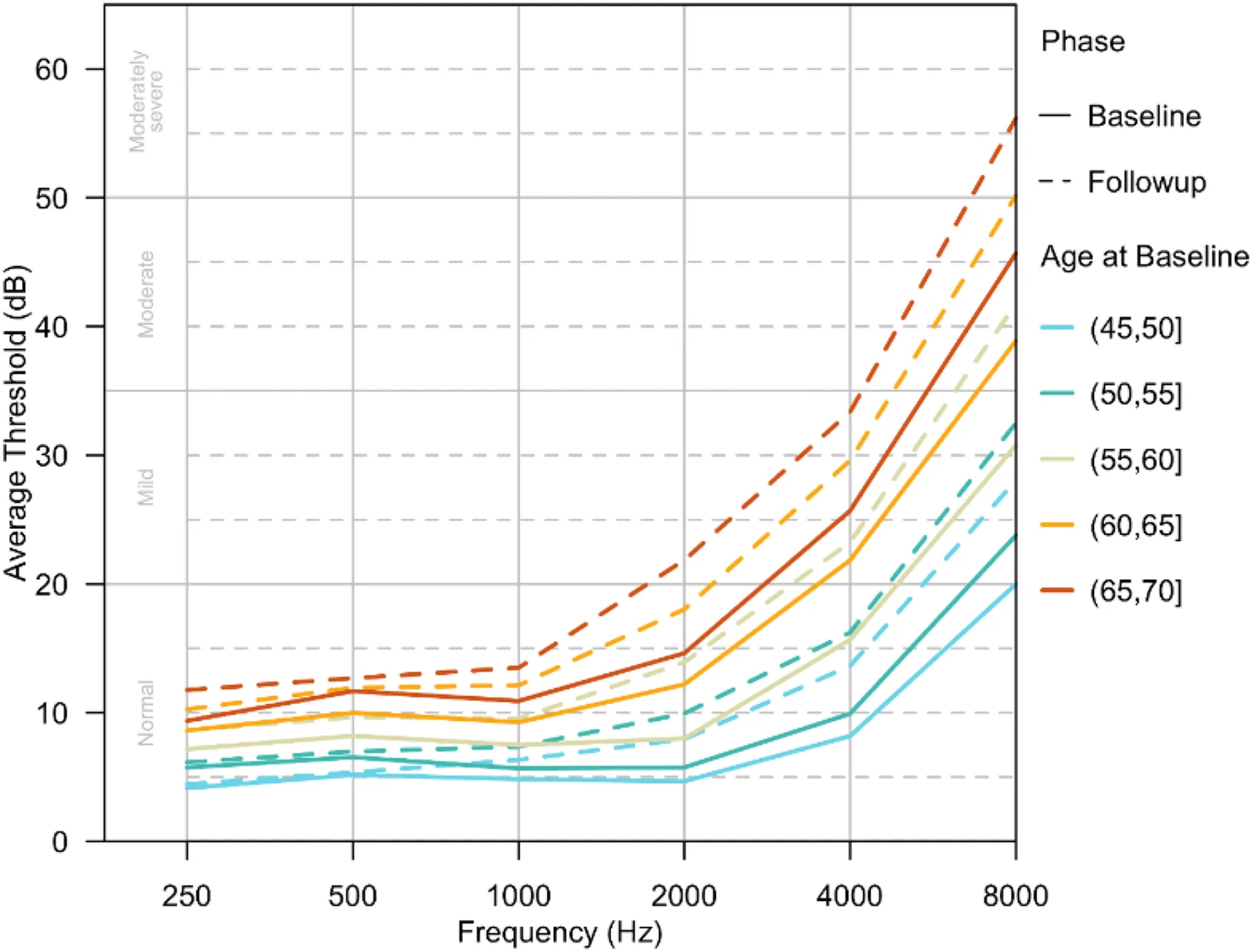
Hearing thresholds by audiometric test frequency of cohort by age group (years) and time of assessment. Solid lines depict baseline values (T0) of hearing loss, and dotted lines depict follow-up (T1) values of hearing loss.

**Fig 2:**
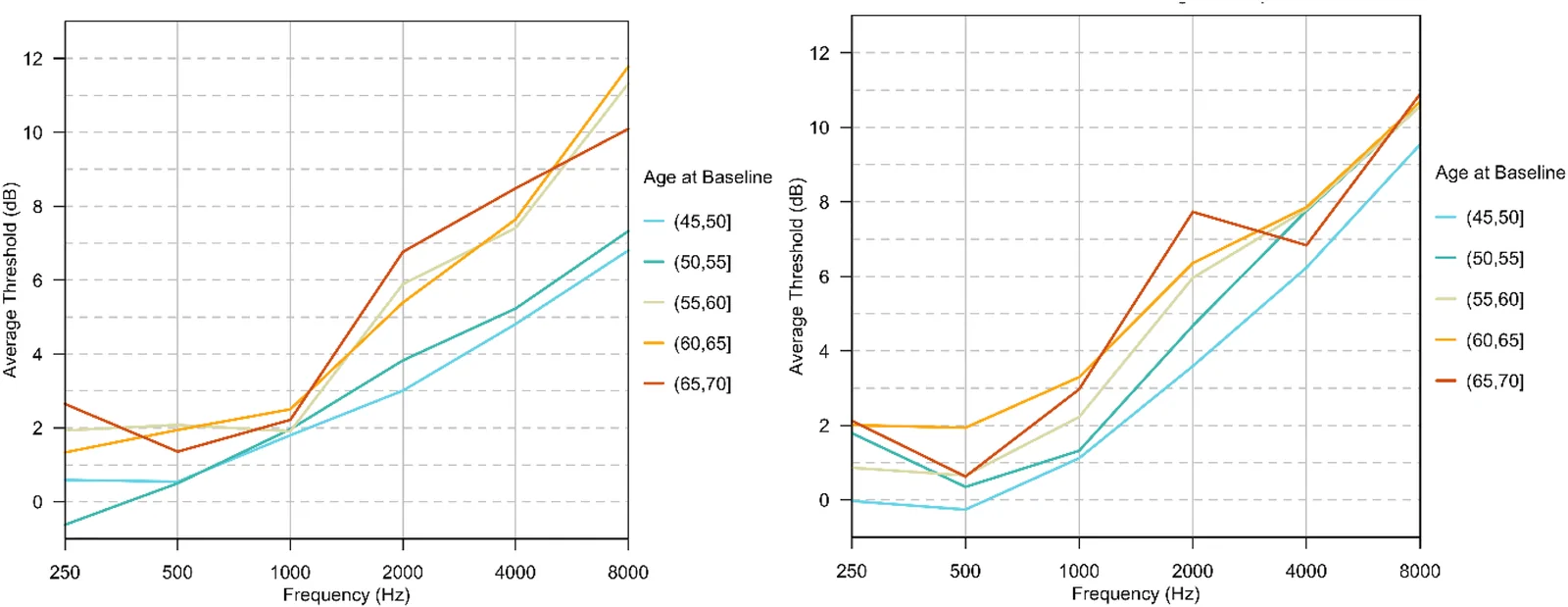
Change in hearing thresholds per frequency between baseline (T0) and follow-up (T1), by age group and sex (left panel: females; right panel: males).

### Hearing outcomes by WHO classification

At baseline (T0) 14% (n = 505) of participants had mild-or-greater hearing loss based on 4FPTA, increasing to 26% (n = 938) at follow-up; for HFPTA, prevalence increased from 53% (n = 1905) to 71% (n = 2557) (Tables 3 and 4). Men consistently had higher rates of hearing loss than women. Among participants with normal 4FPTA at baseline, 14% progressed to at least mild hearing loss over six years, with progression more common in men (16%) than women (2%). For HFPTA, 39% of those with normal high-frequency hearing at baseline developed at least mild high-frequency loss by follow-up.

**Table 3:** Confusion matrix of WHO-classification of hearing loss (4FPTA).

4FPTA		Classification T1						
	Classification T0	Normal N(%)	Mild N(%)	Moderate N(%)	Moderately Severe N(%)	Severe N(%)	Profound N(%)	Total N(%)
Females (n = 1987)	Normal	1558(78%)	213(11%)	9(0%)	3(0%)	9(0%)	0	1602(81%)
	Mild	9(0%)	116(6%)	42(2%)	0	2(0%)	1(0%)	170(9%)
	Moderate	0	1(0%)	13(0%)	0	6(0%)	0	20(1%)
	Moderately severe	1(0%)	0	0	2(0%)	1(0%)	0	4(0%)
	Severe	0	0	0	0	0	1(0%)	1(0%)
	Profound	0	0	0	0	0	0	0
Males (n = 1609)	Normal	1079(67%)	210(13%)	8(0%)	2(0%)	0	0	1299(81%)
	Mild	10(1%)	200(12%)	52(3%)	0	2(0%)	0	264(16%)
	Moderate	1(0%)	0	29(2%)	0	9(1%)	0	39(2%)
	Moderately severe	0	0	1(0%)	1(0%)	3(0%)	0	5(0%)
	Severe	0	0	0	1(0%)	0	1(0%)	2(0%)
	Profound	0	0	0	0	0	0	0
All (n = 3596)	Normal	2637(73%)	423(12%)	17(0%)	9(0%)	5(0%)	0	3091(86%)
	Mild	19(1%)	316(9%)	94(3%)	4(0%)	0	1(0%)	434(12%)
	Moderate	1(0%)	1(0%)	42(1%)	15(0%)	0	0	59(2%)
	Moderately severe	1(0%)	0	1(0%)	4(0%)	3(0%)	0	9(0%)
	Severe	0	0	0	0	1(0%)	2(0%)	3(0%)
	Profound	0	0	0	0	0	0	0
	*Total*	2658(74%)	740(21%)	154(4%)	32(1%)	9(0%)	3(0%)	

Confusion matrix of WHO-classification of hearing loss based on 4FPTA hearing levels of participants by sex and overall at baseline (T0) and follow-up (T1). Cells depict numbers of participants within the particular group (and % of the total per sex group).

**Table 4:** Confusion matrix of WHO-classification of high frequency hearing loss.

HF PTA		Classification T1						
	Classification T0	Normal N(%)	Mild N(%)	Moderate N(%)	Moderately Severe N(%)	Severe N(%)	Profound N(%)	Total N(%)
Females (n = 1987)	Normal	730(37%)	371(19%)	52(3%)	9(0%)	1(0%)	8(0%)	1171(59%)
	Mild	19(1%)	213(11%)	224(11%)	26(1%)	3(0%)	3(0%)	488(25%)
	Moderate	1(0%)	3(0%)	106(5%)	109(5%)	10(1%)	2(0%)	231(12%)
	Moderately severe	0	1(0%)	5(0%)	49(2%)	26(1%)	2(0%)	83(4%)
	Severe	0	0	0	1(0%)	10(1%)	1(0%)	12(1%)
	Profound	0	0	1(0%)	0	0	1(0%)	2(0%)
Males (n = 1609)	Normal	269(17%)	207(13%)	37(2%)	6(0%)	1(0%)	0	520(32%)
	Mild	20(1%)	162(10%)	225(14%)	52(3%)	2(0%)	0	461(29%)
	Moderate	0	7(0%)	122(8%)	175(11%)	25(2%)	2(0%)	331(21%)
	Moderately severe	0	1(0%)	4(0%)	120(7%)	87(5%)	4(0%)	216(13%)
	Severe	0	1(0%)	1(0%)	8(0%)	54(3%)	8(0%)	72(4%)
	Profound	0	0	0	0	1(0%)	8(0%)	9(1%)
All (n = 3596)	Normal	999(28%)	578(16%)	89(2%)	15(0%)	2(0%)	8(0%)	1691(47%)
	Mild	39(1%)	375(10%)	449(12%)	78(2%)	5(0%)	3(0%)	949(26%)
	Moderate	1(0%)	10(0%)	228(6%)	284(8%)	35(1%)	4(0%)	562(16%)
	Moderately severe	0	2(0%)	9(0%)	169(5%)	113(3%)	6(0%)	299(8%)
	Severe	0	1(0%)	1(0%)	9(0%)	64(2%)	9(0%)	84(2%)
	Profound	0	0	1(0%)	0	1(0%)	9(0%)	11(0%)
	*Total*	1039(29%)	966(27%)	777(22%)	555(15%)	220(6%)	39(1%)	

Confusion matrix of WHO-classification of hearing loss based on high frequency PTA (HFPTA) hearing levels of participants by sex and overall at baseline (T0) and follow-up (T1). Cells depict numbers of participants within the particular group (and % of the total per (sex) group).

Of the 3091 participants with a normal hearing level 4FPTA at baseline (T0), 433 (14%) progressed to at least a mild hearing loss at follow-up (T1). This was 2% for females (n = 34) and 16% for males (n = 209) (Table 3).

Of the 1691 participants with a normal hearing level HFPTA at baseline (T0), 652 (39%) progressed to a at least a significant hearing loss at follow-up (T1). This was 36% for females (n = 421) and 41% for males (n = 211) (Table 4).

### Outcomes of univariate linear mixed models of factors associated with change in hearing

In univariate linear mixed models, several factors were associated with hearing deterioration in 4FPTA between T0 and T1 (Table 5). Each additional year of age was associated with a 0.65 dB worsening (95% CI 0.62–0.67), and men showed, on average, 3.16 dB greater decline than women (95% CI 2.52–3.80). Higher BMI, smoking pack-years, elevated or hypertensive blood pressure, pre-diabetes and diabetes, cardiovascular disease, tinnitus, chronic ear infection, and working in a noisy workplace (with inconsistent or no hearing protection) were all associated with worsening in hearing thresholds compared with their respective reference groups (See Table 5). Most of these associations remained significant after adjustments for age and sex, except for elevated bloodpressure, cardiovascular disease and having diabetes (See Table 5).

**Table 5:** Associations between factors and change of hearing levels.

Variable	Effect β1 (95%)	Adjusted effect β1 (95%)		Variable	Effect β1 (95%)	Adjusted effect β1 (95%)
Demographics				General health		
Age (year)	0.65 (0.62-0.67)	0.62 (0.52–0.72)		Blood pressure		
Sex				Normal	Ref	
Female	Ref			Elevated	1.18 (0.22-2.15)	0.24 (−0.66-1.13)
Male	3.16 (2.52–3.80)	2.94 (2.35–3.54)		Hypertension I	3.04 (2.23-3.85)	0.86 (0.10-1.63)
Ear-related health				Hypertension II	3.90 (2.80-5.00)	1.19 (0.17-2.22)
Noisy exposure				BMI (kg/m^2)	2.13 (1.55–2.71)	0.93 (0.41–1.46)
Quiet workplace	Ref			Cardiovascular diseases		
Noisy-workplace-Always protected	−0.56 (−1.54–0.42)	−0.59 (−1.46–0.27)		No	Ref	
Noisy-workplace- Frequently protected	0.87 (0.13–1.61)	0.35 (−0.32–1.02)		Yes	1.18 (0.75–1.61)	0.07 (−0.30-0.44)
Noisy-workplace- Occasionally protected	1.20 (0.60–1.81)	1.02 (0.47–1.57)		Diabetes		
Noisy-workplace-Never protected	1.43 (0.86–2.00)	0.73 (0.24–1.22)		No	Ref	
Tinnitus				Pre-DM	1.82 (1.16-2.47)	0.93 (0.33-1.54)
No	Ref			DM	2.50 (0.82-4.19)	0.72 (−0.83-2.26)
Yes	3.30 (2.80–3.80)	1.70 (1.26–2.14)		Smoking (pack-year)	2.13 (1.72–2.54)	0.81 (0.45–1.17)
Chronic ear infection				General health score		
No	Ref			Poor	Ref	
Yes	0.95 (0.16–1.74)	0.88 (0.20–1.56)		Fair	−0.89 (−2.88–1.10)	−0.20 (−1.89–1.49)
				Good	−1.21 (−3.19–0.76)	−0.13 (−1.81–1.55)
				Very good	−1.59 (−3.57–0.39)	−0.30 (−1.99–1.40)
				Excellent	−2.72 (−4.76–-0.67)	−0.54 (−2.28–1.21)

Outcomes of linear mixed model effects (unadjusted and adjusted analysis for age and sex) to assess associations between factors and change of hearing levels providing outcomes of univariate models (β1, 95% CI) and their effects on 4F PTA hearing levels change over time between baseline(T0) and follow-up (T1).

## Discussion

In this longitudinal population-based study of middle-aged adults, hearing thresholds declined significantly over six years, and several demographics, ear-related, metabolic, and cardiovascular factors were associated with hearing deterioration. Beyond age and sex, higher BMI, occasionally or absent protection in noisy workplaces, tinnitus, chronic ear infection, pre-diabetes and hypertension were all linked to decline in hearing sensitivity after adjustments for age and sex.

Over six years, 14% of those with normal hearing at baseline progressed to a least a mild hearing loss according to the WHO classification. This was 2% for females and 16% for males. These numbers are in line with outcomes of previous population-based cohort studies such as the Epidemiology of Hearing loss study (EHLS; Beaver Dam study) including adults aged 48–92 years at baseline, reporting a 5-year incidence of hearing impairment of 21% [11].

As expected, high-frequency hearing loss was far more common than mid-frequency loss, with prevalence rising from 53% to 71% over six years. Being male compared to females was statistically significantly associated with hearing deterioration over time during the six-year interval with rates of 16% versus 2% respectively of those progressing from no to at least a mild mid-frequency hearing loss. However, in the high-frequencies males and females showed similar rates of those progressing from no to at least a mild hearing loss (in males in 41%, in females in 36%). This has also been reported in previous population-based cohort-studies such as the Canadian Longitudinal Study of Ageing [14], and from the Australian Dynamic Analyses to Optimise Ageing (DYNOPTA) project [6]; in these studies the findings were explained by lower initial hearing levels for men, reflecting an earlier age onset of hearing loss, with females progressing to similar levels of hearing loss as males at a later stage in life. This also appears to be true for our study, particularly at higher frequencies. Therefore, future studies could focus on investigating how differences in sex influence incident hearing loss and hearing change over, ideally by including even younger participants [14].

This study extends previous population/based studies, in that we not only included demographic or general health factors, but also ear-related factors to analyze their involvement in hearing change over time. The finding that a history of chronic ear infection is associated with hearing deterioration, supports clinical knowledge that chronic ear infections have negative consequences to hearing levels [26]. This can be explained by the fact that an inflammatory process can result in permanent changes to the middle ear mechanics creating a conductive hearing loss [27]. Furthermore, recurrent or chronic otitis media are generally considered to contribute to sensorineural hearing loss due to the harmful effects of middle-ear inflammatory toxins on the inner ear function [28]. However, evidence from observational studies supporting these associations remains weak. In a Norwegian population-based cohort study of 32,786 participants who had their hearing tested by pure-tone audiometry in primary school and again at ages ranging from 20–56 years, children with recurrent or chronic otitis media showed reduced adult hearing loss compared to children without infections [29]. In the current study we confirmed that a history of chronic ear infection was associated with hearing deterioration over an observation period of approximately six years in this cohort of adults aged 45–70. It is important to note that the identification of chronic ear infection history was based on self-report, without quantification of the frequency, type or severity of the chronic infection. Prospective studies incorporating detailed assessment of these variables would enable in-depth analysis of the association between otitis media and its long-term effects on hearing.

Another noteworthy aspect of our study is the classification of workplace noise exposure in combination with the level of hearing protection used by participants. Our analysis indicates that working in a noisy workplace while only occasionally or never using hearing protection is associated with deterioration over time. Findings across population-based studies, however, have been inconsistent. For instance, Cruickshanks et al. (EHLS study, 2010) found that a history of a noisy occupation was not associated with the 10-year incidence of hearing impairment [10], Linssen et al (Maastricht Aging Study, 2014) identified a statistically significant association between manual occupation and faster hearing deterioration [13]. This contrasts with the findings of Kiely et al. (DYNOPTA project, 2012), who reported that self-reported workplace noise exposure was associated with baseline levels of hearing loss, but did not predict changes in hearing thresholds over an 11-year observation period [6]. These inconsistencies could be related to the methods used to identify noise exposure. By adding information on used hearing protection, we were able to specify this in more detail which adds value to current knowledge on this topic. However, the present study and those cited are all subject to inaccuracies inherent in self-reporting historical noise exposure and use of hearing protection.

Similarly, differences in methods across studies could explain contradictory results on the relationships between cardiometabolic factors and hearing change in population-based studies. To be noted, hypertension [6,14], cardiovascular risk profile [14], poorly controlled DM [9] and smoking [9,14] have previously all been found to be associated with greater loss of hearing over time, whilst in other studies these factors were not confirmed [15,16]. In our study, having hypertension, a higher BMI and a pre-diabetic status at baseline, were found to be a statistically significant risk factor for deterioration of hearing over time after adjusting for age and sex. For this, we relied on physical and biochemistry measures to identify these factors at baseline. By this the effect of medication to counteract a known or already diagnosed hypertensive or hyperglycemic status was accounted for. However, having a diabetic status at baseline turned out to be no longer a statistically significant risk factor for deterioration of hearing over time after adjusting for age and sex. To date, the exact underlying mechanisms and the temporal relationship between exposure to these factors and onset of hearing loss are not clear. Moreover, strong conclusions about the relationship between cardiovascular and metabolic status and hearing loss are hindered by the cumulative effects of ageing, noise exposure, ototoxic drugs, lifestyle amongst others [30].

The strength of the current study is the large study sample of middle-aged people from the general population with high participation rates and with a wide range of potential risk factors related to demographics, general and ear-related health. Secondly, although information on general health status, noise exposure, and medical history relied on self-report which is subject of reporting bias [31], an important strength of this study is that for other measures this study relied on objective assessments of hearing levels as well as on general health measures such as blood pressure and blood glucose levels [31] Additionally, once individuals are aware of their disease or high cardiometabolic risk profile, one could expect that glycemic, lipid and blood pressure control would be initiated or recommended by their physician. This could influence the observed effect of having the disease on the outcome. Although this potential bias is reduced by relying on objective measurements, in our study disease classification was still based on a single baseline value, which may limit accuracy. On the other hand, non-adherence to prescribed medication for diabetes or cardiovascular diseases is well documented and associated with inadequate disease control [32,33] emphasizing the importance of using objective measures for these factors when drawing conclusions.

Some methodological limitations need to be considered. First, for the study we only assessed the records of 3679 participants of the initial 5107 who initially agreed to the study, which was mainly related to having no visit to the testing center at time of apportionment and thereby having no hearing assessment outcomes leading to exclusion. Comparison of the demographic characteristics of included and excluded participants on baseline characteristics of age and sex did not reveal any meaningful differences. Furthermore, only 61 (1.7%) of the remaining 3657 participants were excluded from analysis based on insufficient hearing test quality. Based on these findings, we consider the potential impact of selective exclusion on the study outcomes to be limited*.* Secondly, although a large and broad sample of adults aged 45–70 years was included, the younger age group between 45 and 50 years of age and males were slightly underrepresented compared to population statistics [24]. Thirdly, we based our risk analysis on calculations of hearing levels of the better ear, which could influence results. For example, the effect of noise exposure on hearing outcomes may vary differently between the two ears, potentially leading to an underestimation of the overall effect. Fourthly, we based our outcomes on the difference in hearing levels between two assessments, about six years apart. A longer follow-up could provide additional inside on risk factors or risk factor assessment by sex. Therefore, studies with longer term follow-up data are warranted. Finally, we used measures of health-related variables at baseline in relation to change in hearing over time. As a result, any influence of changes in health status during follow-up on hearing decline was not considered. This reflects clinical practice, where baseline health characteristics are often the information available when evaluating an individual and discussing their expected hearing trajectory. Examining the impact of changes in health status over time could be of interest for future studies.

Considering the high prevalence of hearing loss by age and their impact on quality of daily life of those affected, further research is needed to determine the underlying mechanisms and relationships between demographic, ear-related and environmental factors and hearing in the long term. Therefore, additional population-based data studies detailing this information and including data of long-term follow-up are needed to clarify these relationships. The current information on factors associated with hearing deterioration can be used by clinicians when counselling their patients about their hearing loss, and for developing public health policy particularly around common conditions such as hypertension and obesity or preventable factors such as noise exposure

## Conclusions

In this longitudinal population-based study of 3596 middle-aged Western Australians we demonstrated that hearing thresholds significantly declined over a six-year period, with 14% of those with normal hearing at baseline developing at least mild hearing loss. Beyond age and sex, several modifiable factors (such as a smoking, pre-diabetes, hypertension, a higher BMI and unprotected workplace noise exposure) were independently associated with hearing deterioration over time. These findings underscore the importance of early identification and targeted prevention strategies, particularly in at-risk populations, and highlight the need for future research to further clarify the complex interplay between metabolic health, environmental exposures, and hearing outcomes.

## Supporting information

S1 Checklist STROBE Statement—Checklist of items that should be included in reports of *cohort studies.* (PDF)
