# Supplementary material for: Change in hearing loss and its association with general health in a community-based longitudinal study of middle-aged individuals: The Busselton healthy ageing study
Source: PLoS One. 2026 Oct 15;21(10):e0360651. doi: 10.1371/journal.pone.0360651 (PMC13671500; doi:10.1371/journal.pone.0360651)
Supplement: Supplementary file 2 [file pone.0360651.s002.docx]

**Review of the manuscript**

**Title:** Change in Hearing loss and its association with general health in a community-based longitudinal study of middle-aged individuals: the Busselton Healthy Ageing Study

**Summary**

This manuscript presents a longitudinal population-based study investigating the progression of hearing loss and its association with demographic, general health, and environmental factors in middle-aged adults from the Busselton Healthy Ageing Study. The authors analyzed audiometric data collected at baseline and after a six-year follow-up and used linear mixed models to assess associations between various risk factors and changes in hearing thresholds. The study shows that hearing thresholds worsened over time, particularly in men and at higher frequencies, and that several metabolic and environmental factors were associated with faster hearing decline.

Overall, the study addresses an important public health topic and benefits from a longitudinal design and a relatively large sample size. However, there are several methodological concerns that should be addressed before the manuscript can be considered for publication.

**Major Comments**

1. **Use of univariate models only**
   The analysis appears to be based on univariate linear mixed models. Many of the investigated variables (e.g., age, sex, BMI, cardiovascular disease, smoking, hypertension, diabetes) are interrelated, and without multivariable models adjusting for confounding variables, it is difficult to assess the independent association of each factor with hearing decline. Multivariable models should be performed and presented.
2. **Multiple imputation methodology**
   The authors state that multiple imputation with five imputations was performed; however, only the first imputed dataset was used for the analysis. This is not consistent with recommended multiple imputation methodology. Results should be pooled across all imputed datasets using appropriate methods (e.g., Rubin’s rules). This issue should be corrected and analyses repeated.
3. **Potential selection bias**
   A substantial number of participants were excluded from the final analysis due to missing or unreliable audiometry. The authors should provide a comparison of baseline characteristics between included and excluded participants to assess potential selection bias.
4. **Exposure variables measured only at baseline**
   Health-related and environmental variables were measured only at baseline, while hearing outcomes were measured longitudinally. Changes in health status over the follow-up period were not considered, which may influence the observed associations. This limitation should be discussed more clearly.
5. **Data availability**
   The data availability statement indicates that data are available from a third party upon request. PLOS ONE requires that data underlying the findings be fully available whenever possible. The authors should clarify whether the dataset can be deposited in a public repository or provide a stronger justification for restricted access.

**Minor Comments**

1. There is a typographical error in the title (“Change in Hearing los” should be corrected to “loss”).
2. Please clarify whether audiometric equipment was regularly calibrated and how quality control of audiometry was ensured.
3. Several variables (e.g., noise exposure, tinnitus, chronic ear infection, general health) are based on self-report and may be subject to recall bias; this should be acknowledged as a limitation.
4. Please clarify whether interaction effects (e.g., age × sex) were tested.
5. The manuscript states that the study follows the STROBE guidelines; please ensure that the STROBE checklist is included as supplementary material.
6. Please provide more detail on the linear mixed model structure (random effects, covariance structure, and model assumptions).

**Recommendation**

**Major revision**

The study is valuable and based on a large longitudinal dataset, but important methodological and statistical issues need to be addressed before the results can be reliably interpreted.

*REVIEWER*
