# Supplementary material for: Change in hearing loss and its association with general health in a community-based longitudinal study of middle-aged individuals: The Busselton healthy ageing study
Source: PLoS One. 2026 Oct 15;21(10):e0360651. doi: 10.1371/journal.pone.0360651 (PMC13671500; doi:10.1371/journal.pone.0360651)
Supplement: Supplementary file 3 [file pone.0360651.s004.docx]

**Review of the manuscript**

**Title:** Change in Hearing loss and its association with general health in a community-based longitudinal study of middle-aged individuals: the Busselton Healthy Ageing Study

**Summary**

This manuscript presents a longitudinal population-based study investigating the progression of hearing loss and its association with demographic, general health, and environmental factors in middle-aged adults from the Busselton Healthy Ageing Study. The authors analyzed audiometric data collected at baseline and after a six-year follow-up and used linear mixed models to assess associations between various risk factors and changes in hearing thresholds. The study shows that hearing thresholds worsened over time, particularly in men and at higher frequencies, and that several metabolic and environmental factors were associated with faster hearing decline.

Overall, the study addresses an important public health topic and benefits from a longitudinal design and a relatively large sample size. However, there are several methodological concerns that should be addressed before the manuscript can be considered for publication.

**Major Comments**

1. **Use of univariate models only**
   The analysis appears to be based on univariate linear mixed models. Many of the investigated variables (e.g., age, sex, BMI, cardiovascular disease, smoking, hypertension, diabetes) are interrelated, and without multivariable models adjusting for confounding variables, it is difficult to assess the independent association of each factor with hearing decline. Multivariable models should be performed and presented.

We appreciate the reviewer’s comment regarding multivariate adjustment. We have added multivariable adjustment. Our cohort was based on a random sample of the Busselton population and the age and sex distribution of the study sample differed slightly from that of the general Busselton population (Census data from the Australian Bureau of Statistics). Therefore, we performed an additional adjusted analysis for correct for age and sex. We added the background for this to the methods section (marked manuscript pg 8, line 274-276), added the outcomes to Table 4 and updated the discussion with the insights of this analysis (pg 16. Linke461-463).

Of note, during verification of the analyses and additional calculations, we identified an error in the calculation of the cut-offs used for the diabetes mellitus (DM) and blood pressure categories, as well as in the coding of the cardiovascular disease (CVD) outcomes. We corrected these errors and updated the corresponding results in Tables 1 and 4 accordingly. We sincerely regret these errors. Importantly, although some of the numerical results changed following these corrections, they did not materially affect the overall findings or conclusions of the manuscript.

2. **Multiple imputation methodology**
   The authors state that multiple imputation with five imputations was performed; however, only the first imputed dataset was used for the analysis. This is not consistent with recommended multiple imputation methodology. Results should be pooled across all imputed datasets using appropriate methods (e.g., Rubin’s rules). This issue should be corrected and analyses repeated.

Thank you for this comment. We agree that the description of the handling of missing data in the Methods section was insufficiently clear. We have therefore revised this section, which now reads as follows*: Missing data were anticipated in covariates included in subsequent analyses. Missing data were handled using multivariate imputation by chained equations. The mice package in R was used to generate five imputed datasets, using predictive mean matching as the imputation method, a maximum of 10 iterations, and a random seed of 12345. Analyses were performed separately within each imputed dataset, and parameter estimates and standard errors were subsequently pooled across the five imputed datasets using Rubin’s rules (marked manuscript pg 8 line 259-264).*

3. **Potential selection bias**
   A substantial number of participants were excluded from the final analysis due to missing or unreliable audiometry. The authors should provide a comparison of baseline characteristics between included and excluded participants to assess potential selection bias.

We contacted the study center to verify the previously reported numbers of exclusions based on the audiometry quality and other reasons for exclusion, which allowed us to correct and clarify several of the exclusion categories. Importantly, the reduction in the number of participants available for the present analyses was primarily due to participants not attending the study-center visit at both T0 and T1, rather than to missing or unreliable audiometry. Of the 5,107 participants who initially agreed to participate, 3,679 (72%) attended the study center at both time points and completed the physical assessments, including audiometry. Among these participants, 22 records could not be matched between time points, while only 18 participants at T0 and 43 participants at T1 were excluded because of insufficient hearing-test quality. This resulted in a final sample of 3,596 participants. We revised the first paragraph of the Results section to clearly report these numbers and reasons for exclusion (marked manuscript pg 9 line 304-310)

Based on this clarification, we consider potential selection bias related to study-center attendance to be more relevant than bias related to hearing-test quality, as may have been suggested by the previous description. We therefore added non-attendance at the study-center visits as a potential source of selection bias to the limitations section of the Discussion. To further assess this issue, we compared the baseline characteristics of participants included in the present analyses with those of all participants who initially agreed to participate and found no meaningful differences. We therefore found no indication that selection related to study-center attendance materially influenced the study findings, and this explanation has also been added to the Discussion (marked manuscript pg 17, line 485-492)

4. **Exposure variables measured only at baseline**
   Health-related and environmental variables were measured only at baseline, while hearing outcomes were measured longitudinally. Changes in health status over the follow-up period were not considered, which may influence the observed associations. This limitation should be discussed more clearly.

*From a clinical and public health perspective, baseline characteristics often represent the information available when evaluating an individual, and our analyses therefore provide insight into the expected hearing trajectory associated with these characteristics over the following six years. Nevertheless, changes in health-related variables during follow-up may have influenced the observed associations. To acknowledge this issue and its potentially unknown impact on the study outcomes, we have added this point to the limitations section of the Discussion.(marked manuscript pg 17, line 500-505)*

5. **Data availability**
   The data availability statement indicates that data are available from a third party upon request. PLOS ONE requires that data underlying the findings be fully available whenever possible. The authors should clarify whether the dataset can be deposited in a public repository or provide a stronger justification for restricted access.

*We agree that data availability is an important issue. For the current study, data access is permitted under the approval granted by the ethics committee involved, provided that the use of the data complies with the terms and conditions set by the Busselton Population Medical Research Institute. Therefore, we have revised the data availability statement accordingly: Data from the Busselton Health Study are restricted due to ethical and privacy requirements. De-identified datasets may be accessed by external researchers through the Busselton Population Medical Research Institute (BPMRI) following ethics approval and a Data Access Agreement. For access requests, visit* [*https://bpmri.org.au/research/database-access.html*](https://eur05.safelinks.protection.outlook.com/?url=https%3A%2F%2Fbpmri.org.au%2Fresearch%2Fdatabase-access.html&data=05%7C02%7CA.L.Smit-9%40umcutrecht.nl%7Cb8e67d33ab434d3e0d7108df183c5456%7Cdcdf4a3dd0c04a6394cf781981249be5%7C0%7C0%7C639256319557188950%7CUnknown%7CTWFpbGZsb3d8eyJFbXB0eU1hcGkiOnRydWUsIlYiOiIwLjAuMDAwMCIsIlAiOiJXaW4zMiIsIkFOIjoiTWFpbCIsIldUIjoyfQ%3D%3D%7C0%7C%7C%7C&sdata=XjmGyc6Z9CGWft2dJ5ojhyz3iV0AvKBq%2FCAxWsAYkyA%3D&reserved=0) *or contact* [*kevin.murray@uwa.edu.au*](mailto:kevin.murray@uwa.edu.au)*.*

**Minor Comments**

1. There is a typographical error in the title (“Change in Hearing los” should be corrected to “loss”).

*Corrected accordingly*

2. Please clarify whether audiometric equipment was regularly calibrated and how quality control of audiometry was ensured.

*Quality of the audiometric equipment was maintained by (i) training of the technical staff, and (ii) the use of QUALIND which monitors false positives and response times, and flags audiogram as POOR or FAIL if they are not reliable. This was built into the automated audiometer software. We added this information and references on its background to the methods section (marked manuscript pg 5-6, line 180-189).*

3. Several variables (e.g., noise exposure, tinnitus, chronic ear infection, general health) are based on self-report and may be subject to recall bias; this should be acknowledged as a limitation.

*We addressed this issue in the part in the discussion on chronic ear infections, but now mentioned this more specifically in the discussion part on the limitation. (pg 16 line 471-476)*

4. Please clarify whether interaction effects (e.g., age × sex) were tested.

*These were not intended and not tested in line with the answer provided under major comments bullet 1.*

5. The manuscript states that the study follows the STROBE guidelines; please ensure that the STROBE checklist is included as supplementary material.

*We now included the checklist*

6. Please provide more detail on the linear mixed model structure (random effects, covariance structure, and model assumptions).

*We have revised the methods section on this and provided the additional information (marked manuscript pg 8 line 270-289).*

**Recommendation**

**Major revision**

The study is valuable and based on a large longitudinal dataset, but important methodological and statistical issues need to be addressed before the results can be reliably interpreted.
