# Supplementary material for: Change in hearing loss and its association with general health in a community-based longitudinal study of middle-aged individuals: The Busselton healthy ageing study
Source: PLoS One. 2026 Oct 15;21(10):e0360651. doi: 10.1371/journal.pone.0360651 (PMC13671500; doi:10.1371/journal.pone.0360651)
Supplement: Peer review history

# Peer review: Decision Letter 0

**DOI:** 10.1371/journal.pone.0360651.r001

**Authors:** Gauri Mankekar

4 Apr 2026

Dear Dr. Smit,

Thank you for submitting your manuscript to PLOS ONE. After careful consideration, we feel that it has merit but does not fully meet PLOS ONE’s publication criteria as it currently stands. Therefore, we invite you to submit a revised version of the manuscript that addresses the points raised during the review process.

==============================

**ACADEMIC EDITOR: Please see comments of reviewer and revise your manuscript before re-submission.**

==============================

Please submit your revised manuscript by May 19 2026 11:59PM. If you will need more time than this to complete your revisions, please reply to this message or contact the journal office at plosone@plos.org. When you're ready to submit your revision, log on to https://www.editorialmanager.com/pone/ and select the 'Submissions Needing Revision' folder to locate your manuscript file.

- A letter that responds to each point raised by the academic editor and reviewer(s). You should upload this letter as a separate file labeled 'Response to Reviewers'.
- A marked-up copy of your manuscript that highlights changes made to the original version. You should upload this as a separate file labeled 'Revised Manuscript with Track Changes'.
- An unmarked version of your revised paper without tracked changes. You should upload this as a separate file labeled 'Manuscript'.

If you would like to make changes to your financial disclosure, please include your updated statement in your cover letter. Guidelines for resubmitting your figure files are available below the reviewer comments at the end of this letter.

If applicable, we recommend that you deposit your laboratory protocols in protocols.io to enhance the reproducibility of your results. Protocols.io assigns your protocol its own identifier (DOI) so that it can be cited independently in the future. For instructions see: https://journals.plos.org/plosone/s/submission-guidelines#loc-laboratory-protocols. Additionally, PLOS ONE offers an option for publishing peer-reviewed Lab Protocol articles, which describe protocols hosted on protocols.io. Read more information on sharing protocols at https://plos.org/protocols?utm_medium=editorial-email&utm_source=authorletters&utm_campaign=protocols.

As the corresponding author, your ORCID iD is verified in the submission system and will appear in the published article. PLOS supports the use of ORCID, and we encourage all coauthors to register for an ORCID iD and use it as well. Please encourage your coauthors to verify their ORCID iD within the submission system before final acceptance, as unverified ORCID iDs will not appear in the published article. *Only*  the individual author can complete the verification step; PLOS staff *cannot*  verify ORCID iDs on behalf of authors.

We look forward to receiving your revised manuscript.

Kind regards,

Gauri Mankekar, MD,PhD,FACS

Academic Editor

PLOS One

Journal Requirements:

When submitting your revision, we need you to address these additional requirements.

1. Please ensure that your manuscript meets PLOS ONE's style requirements, including those for file naming. The PLOS ONE style templates can be found at

https://journals.plos.org/plosone/s/file?id=wjVg/PLOSOne_formatting_sample_main_body.pdf and https://journals.plos.org/plosone/s/file?id=ba62/PLOSOne_formatting_sample_title_authors_affiliations.pdf

2. We note that you have indicated that there are restrictions to data sharing for this study. For studies involving human research participant data or other sensitive data, we encourage authors to share de-identified or anonymized data. However, when data cannot be publicly shared for ethical reasons, we allow authors to make their data sets available upon request. For information on unacceptable data access restrictions, please see http://journals.plos.org/plosone/s/data-availability#loc-unacceptable-data-access-restrictions.

Before we proceed with your manuscript, please address the following prompts:

a) If there are ethical or legal restrictions on sharing a de-identified data set, please explain them in detail (e.g., data contain potentially identifying or sensitive patient information, data are owned by a third-party organization, etc.) and who has imposed them (e.g., a Research Ethics Committee or Institutional Review Board, etc.). Please also provide contact information for a data access committee, ethics committee, or other institutional body to which data requests may be sent.

b) If there are no restrictions, please upload the minimal anonymized data set necessary to replicate your study findings to a stable, public repository and provide us with the relevant URLs, DOIs, or accession numbers. Please see http://www.bmj.com/content/340/bmj.c181.long for guidelines on how to de-identify and prepare clinical data for publication. For a list of recommended repositories, please see https://journals.plos.org/plosone/s/recommended-repositories. You also have the option of uploading the data as Supporting Information files, but we would recommend depositing data directly to a data repository if possible.

Please update your Data Availability statement in the submission form accordingly.

3. We note that the grant information you provided in the ‘Funding Information’ and ‘Financial Disclosure’ sections do not match.

When you resubmit, please ensure that you provide the correct grant numbers for the awards you received for your study in the ‘Funding Information’ section.

4. Please amend either the title on the online submission form (via Edit Submission) or the title in the manuscript so that they are identical.

5. If the reviewer comments include a recommendation to cite specific previously published works, please review and evaluate these publications to determine whether they are relevant and should be cited. There is no requirement to cite these works unless the editor has indicated otherwise.

6. Please review your reference list to ensure that it is complete and correct. If you have cited papers that have been retracted, please include the rationale for doing so in the manuscript text, or remove these references and replace them with relevant current references. Any changes to the reference list should be mentioned in the rebuttal letter that accompanies your revised manuscript. If you need to cite a retracted article, indicate the article’s retracted status in the References list and also include a citation and full reference for the retraction notice.

[Note: HTML markup is below. Please do not edit.]

Reviewers' comments:

Reviewer's Responses to Questions

**Comments to the Author**

1. Is the manuscript technically sound, and do the data support the conclusions?

Reviewer #1: Yes

Reviewer #2: Yes

**********

2. Has the statistical analysis been performed appropriately and rigorously?

Reviewer #1: Yes

Reviewer #2: No

**********

3. Have the authors made all data underlying the findings in their manuscript fully available?

The PLOS Data policy

Reviewer #1: Yes

Reviewer #2: No

**********

4. Is the manuscript presented in an intelligible fashion and written in standard English?

Reviewer #1: Yes

Reviewer #2: Yes

**********

Reviewer #1: It is an ambitious study with many variables. Chronic ear infections are known to cause hearing loss. None of these patients were examined clinically even though just 6% claimed of chronic ear infections.None of the patients were subjected to a clinical examination. A clinical examination of the ear of the patients included in this study would have added value. Instead, this study relied on self reporting, an unreliable method indeed.

Secondly only the better ear was used for reporting. Another major factor that can distort reporting.

The study has value because of the large number of patients reviewed.

At best this study concludes that there is an association. Not a direct cause and effect relationship.

Reviewer #2: This manuscript presents a longitudinal population-based study investigating the progression of hearing loss and its association with demographic, general health, and environmental factors. The longitudinal design, relatively large sample size, and use of objective audiometric measurements are important strengths of the study.

However, there are several methodological issues that should be addressed. First, the analysis appears to be based mainly on univariate models, and multivariable models adjusting for potential confounders should be performed. Second, although multiple imputation was conducted, only one imputed dataset was used for the analysis, which is not consistent with recommended multiple imputation methodology. Results should be pooled across all imputed datasets. Third, a comparison between included and excluded participants should be provided to assess potential selection bias. In addition, health-related variables were measured only at baseline, and changes over time were not considered, which should be discussed as a limitation.

The data availability statement should also be clarified, as PLOS ONE requires that data underlying the findings be fully available whenever possible.

Overall, this is an interesting and potentially valuable longitudinal study; however, the methodological and statistical issues should be addressed before the manuscript can be considered for publication. I recommend major revision.

**********

what does this mean?). If published, this will include your full peer review and any attached files.

If you choose “no”, your identity will remain anonymous but your review may still be made public.

**Do you want your identity to be public for this peer review?**  For information about this choice, including consent withdrawal, please see our Privacy Policy

Reviewer #1: **Yes:**  Professor Christopher de Souza MS, DORL, DNB, FACS FRCS(E), FRCS (I), FRCP (E), D.Litt HC

Reviewer #2: No

**********

[NOTE: If reviewer comments were submitted as an attachment file, they will be attached to this email and accessible via the submission site. Please log into your account, locate the manuscript record, and check for the action link "View Attachments". If this link does not appear, there are no attachment files.]

To ensure your figures meet our technical requirements, please review our figure guidelines: https://journals.plos.org/plosone/s/figures

You may also use PLOS’s free figure tool, NAAS, to help you prepare publication quality figures: https://journals.plos.org/plosone/s/figures#loc-tools-for-figure-preparation.

NAAS will assess whether your figures meet our technical requirements by comparing each figure against our figure specifications.

Attachment Submitted filename: PLOS ONE REVIEW REPORT.docx

# Author comment: Author response to Decision Letter 1

**DOI:** 10.1371/journal.pone.0360651.r002

23 Sep 2026

Review of the manuscript

Title: Change in Hearing loss and its association with general health in a community-based longitudinal study of middle-aged individuals: the Busselton Healthy Ageing Study

Summary

This manuscript presents a longitudinal population-based study investigating the progression of hearing loss and its association with demographic, general health, and environmental factors in middle-aged adults from the Busselton Healthy Ageing Study. The authors analyzed audiometric data collected at baseline and after a six-year follow-up and used linear mixed models to assess associations between various risk factors and changes in hearing thresholds. The study shows that hearing thresholds worsened over time, particularly in men and at higher frequencies, and that several metabolic and environmental factors were associated with faster hearing decline.

Overall, the study addresses an important public health topic and benefits from a longitudinal design and a relatively large sample size. However, there are several methodological concerns that should be addressed before the manuscript can be considered for publication.

Major Comments

1. Use of univariate models only

The analysis appears to be based on univariate linear mixed models. Many of the investigated variables (e.g., age, sex, BMI, cardiovascular disease, smoking, hypertension, diabetes) are interrelated, and without multivariable models adjusting for confounding variables, it is difficult to assess the independent association of each factor with hearing decline. Multivariable models should be performed and presented.

We appreciate the reviewer’s comment regarding multivariate adjustment. We have added multivariable adjustment. Our cohort was based on a random sample of the Busselton population and the age and sex distribution of the study sample differed slightly from that of the general Busselton population (Census data from the Australian Bureau of Statistics). Therefore, we performed an additional adjusted analysis for correct for age and sex. We added the background for this to the methods section (marked manuscript pg 8, line 274-276), added the outcomes to Table 4 and updated the discussion with the insights of this analysis (pg 16. Linke461-463).

Of note, during verification of the analyses and additional calculations, we identified an error in the calculation of the cut-offs used for the diabetes mellitus (DM) and blood pressure categories, as well as in the coding of the cardiovascular disease (CVD) outcomes. We corrected these errors and updated the corresponding results in Tables 1 and 4 accordingly. We sincerely regret these errors. Importantly, although some of the numerical results changed following these corrections, they did not materially affect the overall findings or conclusions of the manuscript.

2. Multiple imputation methodology

The authors state that multiple imputation with five imputations was performed; however, only the first imputed dataset was used for the analysis. This is not consistent with recommended multiple imputation methodology. Results should be pooled across all imputed datasets using appropriate methods (e.g., Rubin’s rules). This issue should be corrected and analyses repeated.

Thank you for this comment. We agree that the description of the handling of missing data in the Methods section was insufficiently clear. We have therefore revised this section, which now reads as follows: Missing data were anticipated in covariates included in subsequent analyses. Missing data were handled using multivariate imputation by chained equations. The mice package in R was used to generate five imputed datasets, using predictive mean matching as the imputation method, a maximum of 10 iterations, and a random seed of 12345. Analyses were performed separately within each imputed dataset, and parameter estimates and standard errors were subsequently pooled across the five imputed datasets using Rubin’s rules (marked manuscript pg 8 line 259-264).

3. Potential selection bias

A substantial number of participants were excluded from the final analysis due to missing or unreliable audiometry. The authors should provide a comparison of baseline characteristics between included and excluded participants to assess potential selection bias.

We contacted the study center to verify the previously reported numbers of exclusions based on the audiometry quality and other reasons for exclusion, which allowed us to correct and clarify several of the exclusion categories. Importantly, the reduction in the number of participants available for the present analyses was primarily due to participants not attending the study-center visit at both T0 and T1, rather than to missing or unreliable audiometry. Of the 5,107 participants who initially agreed to participate, 3,679 (72%) attended the study center at both time points and completed the physical assessments, including audiometry. Among these participants, 22 records could not be matched between time points, while only 18 participants at T0 and 43 participants at T1 were excluded because of insufficient hearing-test quality. This resulted in a final sample of 3,596 participants. We revised the first paragraph of the Results section to clearly report these numbers and reasons for exclusion (marked manuscript pg 9 line 304-310)

Based on this clarification, we consider potential selection bias related to study-center attendance to be more relevant than bias related to hearing-test quality, as may have been suggested by the previous description. We therefore added non-attendance at the study-center visits as a potential source of selection bias to the limitations section of the Discussion. To further assess this issue, we compared the baseline characteristics of participants included in the present analyses with those of all participants who initially agreed to participate and found no meaningful differences. We therefore found no indication that selection related to study-center attendance materially influenced the study findings, and this explanation has also been added to the Discussion (marked manuscript pg 17, line 485-492)

4. Exposure variables measured only at baseline

Health-related and environmental variables were measured only at baseline, while hearing outcomes were measured longitudinally. Changes in health status over the follow-up period were not considered, which may influence the observed associations. This limitation should be discussed more clearly.

From a clinical and public health perspective, baseline characteristics often represent the information available when evaluating an individual, and our analyses therefore provide insight into the expected hearing trajectory associated with these characteristics over the following six years. Nevertheless, changes in health-related variables during follow-up may have influenced the observed associations. To acknowledge this issue and its potentially unknown impact on the study outcomes, we have added this point to the limitations section of the Discussion.(marked manuscript pg 17, line 500-505)

5. Data availability

The data availability statement indicates that data are available from a third party upon request. PLOS ONE requires that data underlying the findings be fully available whenever possible. The authors should clarify whether the dataset can be deposited in a public repository or provide a stronger justification for restricted access.

We agree that data availability is an important issue. Therefore, we have revised the data availability statement accordingly: Data from the Busselton Health Study are restricted due to ethical and privacy requirements. De-identified datasets may be accessed by external researchers through the Busselton Population Medical Research Institute (BPMRI) following ethics approval and a Data Access Agreement. For access requests, visit https://bpmri.org.au/research/database-access.html or contact kevin.murray@uwa.edu.au.

Minor Comments

1. There is a typographical error in the title (“Change in Hearing los” should be corrected to “loss”).

Corrected accordingly

2. Please clarify whether audiometric equipment was regularly calibrated and how quality control of audiometry was ensured.

Quality of the audiometric equipment was maintained by (i) training of the technical staff, and (ii) the use of QUALIND which monitors false positives and response times, and flags audiogram as POOR or FAIL if they are not reliable. This was built into the automated audiometer software. We added this information and references on its background to the methods section (marked manuscript pg 5-6, line 180-189).

3. Several variables (e.g., noise exposure, tinnitus, chronic ear infection, general health) are based on self-report and may be subject to recall bias; this should be acknowledged as a limitation.

We addressed this issue in the part in the discussion on chronic ear infections, but now mentioned this more specifically in the discussion part on the limitation. (pg 16 line 471-476)

4. Please clarify whether interaction effects (e.g., age × sex) were tested.

These were not intended and not tested in line with the answer provided under major comments bullet 1.

5. The manuscript states that the study follows the STROBE guidelines; please ensure that the STROBE checklist is included as supplementary material.

We now included the checklist

6. Please provide more detail on the linear mixed model structure (random effects, covariance structure, and model assumptions).

We have revised the methods section on this and provided the additional information (marked manuscript pg 8 line 270-289).

Recommendation

Major revision

The study is valuable and based on a large longitudinal dataset, but important methodological and statistical issues need to be addressed before the results can be reliably interpreted.

Attachment Submitted filename: PLOS ONE REVIEW REPORT PONE-D-25-68716 12092026 (2) v2.docx

# Editor report: Decision Letter 1

**DOI:** 10.1371/journal.pone.0360651.r003

**Authors:** Gauri Mankekar

30 Sep 2026

Change in Hearing loss and its association with general health in a community-based longitudinal study of middle-aged individuals: the Busselton Healthy Ageing Study

PONE-D-25-68716R1

Dear Dr. Adriana Smit,

We’re pleased to inform you that your manuscript has been judged scientifically suitable for publication and will be formally accepted for publication once it meets all outstanding technical requirements.

Within one week, you’ll receive an e-mail detailing the required amendments. When these have been addressed, you’ll receive a formal acceptance letter and your manuscript will be scheduled for publication.

An invoice will be generated when your article is formally accepted. Please note, if your institution has a publishing partnership with PLOS and your article meets the relevant criteria, all or part of your publication costs will be covered. Please make sure your user information is up-to-date by logging into Editorial Manager at Editorial Manager® and clicking the ‘Update My Information' link at the top of the page. For questions related to billing, please contact billing support.

If your institution or institutions have a press office, please notify them about your upcoming paper to help maximize its impact. If they’ll be preparing press materials, please inform our press team as soon as possible -- no later than 48 hours after receiving the formal acceptance. Your manuscript will remain under strict press embargo until 2 pm Eastern Time on the date of publication. For more information, please contact onepress@plos.org.

Kind regards,

Gauri Mankekar, MD,PhD,FACS

Academic Editor

PLOS One

Additional Editor Comments (optional):

Reviewers' comments:

# Editor report: Acceptance letter

**DOI:** 10.1371/journal.pone.0360651.r004

**Authors:** Gauri Mankekar

PONE-D-25-68716R1

PLOS One

Dear Dr. Smit,

I'm pleased to inform you that your manuscript has been deemed suitable for publication in PLOS One. Congratulations! Your manuscript is now being handed over to our production team.

At this stage, our production department will prepare your paper for publication. This includes ensuring the following:

* All references, tables, and figures are properly cited

* All relevant supporting information is included in the manuscript submission,

* There are no issues that prevent the paper from being properly typeset

You will receive further instructions from the production team, including instructions on how to review your proof when it is ready. Please keep in mind that we are working through a large volume of accepted articles, so please give us a few days to review your paper and let you know the next and final steps.

Lastly, if your institution or institutions have a press office, please let them know about your upcoming paper now to help maximize its impact. If they'll be preparing press materials, please inform our press team within the next 48 hours. Your manuscript will remain under strict press embargo until 2 pm Eastern Time on the date of publication. For more information, please contact onepress@plos.org.

You will receive an invoice from PLOS for your publication fee after your manuscript has reached the completed accept phase. If you receive an email requesting payment before acceptance or for any other service, this may be a phishing scheme. Learn how to identify phishing emails and protect your accounts at https://explore.plos.org/phishing.

If we can help with anything else, please email us at customercare@plos.org.

Thank you for submitting your work to PLOS One and supporting open access.

Kind regards,

PLOS One Editorial Office Staff

on behalf of

Dr. Gauri Mankekar

Academic Editor

PLOS One
